# Comparison of biochemical markers and technetium 99m methoxyisobutylisonitrile imaging in primary and secondary hyperparathyroidism

**DOI:** 10.3389/fendo.2023.1094689

**Published:** 2023-03-27

**Authors:** Yuhua Wang, Ye Liu, Na Li, Wanchun Zhang

**Affiliations:** ^1^ Department of Nuclear Medicine, Shanxi Bethune Hospital, Shanxi Academy of Medical Sciences, Tongji Shanxi Hospital, Third Hospital of Shanxi Medical University, Taiyuan, China; ^2^ Tongji Hospital, Tongji Medical College, Huazhong University of Science and Technology, Wuhan, China

**Keywords:** parathyroid adenoma, parathyroid hyperplasia, primary hyperparathyroidism, secondary hyperparathyroidism, technetium Tc 99m sestamibi, SPECT/CT fusion imaging

## Abstract

**Objective:**

To investigate the differences in biochemical marker levels and the extent of lesion visualization on technetium 99m methoxyisobutylisonitrile (^99m^Tc-MIBI) imaging between primary hyperparathyroidism (PHPT) and secondary hyperparathyroidism (SHPT).

**Methods:**

Nineteen patients with PHPT and 14 patients with SHPT were enrolled in the study, all of whom underwent routine ^99m^Tc-MIBI dual-phase planar imaging, single-photon emission computed tomography combined with computed tomography (SPECT/CT fusion) imaging, and serum biochemical and hormonal investigations prior to surgery. The target-to-non-target (T/NT) ratios were calculated based on images from the early and delayed phases of ^99m^Tc-MIBI planar imaging and also based on SPECT/CT fusion imaging. The volume of the parathyroid glands was measured following their excision.

**Results:**

A total of 62 parathyroid glands were removed: 14 parathyroid adenomas and five parathyroid carcinomas in PHPT patients; and 18 parathyroid adenomas, 17 parathyroid hyperplasia lesions, and eight instances of nodular hyperplasia with adenoma in SHPT patients. The median volume of the lesions in PHPT and SHPT was 1.69 cm^3^ and 0.52 cm^3^ respectively, and the difference between them was statistically significant (*P* = 0.001). The median T/NT ratios calculated at the early phase of ^99m^Tc-MIBI planar imaging, the delayed phase of ^99m^Tc-MIBI planar imaging, and the subsequent SPECT/CT fusion imaging were 1.51, 1.34, and 2.75, respectively, in PHPT, and 1.46, 1.30, and 1.38, in SHPT, respectively. The T/NT ratio difference between PHPT and SHPT on the SPECT/CT fusion imaging was statistically significant (*P* = 0.002). The histopathology subtypes of the lesions were associated with significant differences in two areas: the T/NT ratios on the SPECT/CT fusion imaging and the volume of the lesions (P=0.002, P<0.001).

**Conclusion:**

The proportion of positive findings on ^99m^Tc-MIBI dual-phase planar imaging and the T/NT ratios of ^99m^Tc-MIBI SPECT/CT fusion imaging were higher in PHPT than in SHPT. The volume of parathyroid lesions in SHPT was smaller than in PHPT.

## Introduction

Hyperparathyroidism (HPT) is a generalized disturbance of calcium (Ca) and phosphate metabolism that occurs as a result of the oversecretion of parathyroid hormone (PTH), and it can involve many of the organs and systems within the human body. The underlying cause of the hypersecretion allows HPT to be subdivided into primary hyperparathyroidism (PHPT), secondary hyperparathyroidism (SHPT), and tertiary hyperthyroidism (THPT) (1). The majority of PHPT cases (95%) occur sporadically, approximately 85% of PHPT patients is caused by a solitary adenoma, and about 15-20% are caused by multiple gland disease (2, 3). Hyperfunctional parathyroid glands overproduce PTH and leads to hypercalcemia. SHPT is one of the most common serious complications in patients with chronic renal failure on long-term hemodialysis. Parathyroid cell proliferation and PTH secretion increased is due to persistent hyperphosphatemia, hypocalcemia and a lack of VD. Most SHPT patients have multiple enlarged parathyroid glands and frequent parathyroid anatomical variations in location (4).The clinical manifestations of HPT are diverse and include urinary calculi, osteopenia, pathological fractures, and skeletal deformity.

The main treatment for PHPT is parathyroidectomy. In the treatment of SHPT, vitamin D receptor (VDRA) and calcium supplement are first used to regulate the treatment. If the drug treatment is not effective, surgical resection of the parathyroid gland can be chosen (5). Due to less operative time and fewer complications than traditional bilateral exploration, minimally invasive parathyroidectomy (MIP) is recommended in HPT (6). The anatomical location of parathyroid glands has a higher probability of variation (7). Therefore, accurate localization before surgery can improve the success rate of surgery. There are three different options, subtotal parathyroidectomy, total parathyroidectomy with or without auto-transplantation in SHPT patients. If the surgeon does not remove all hyperfunctioning parathyroid tissue, the recurrent SHPT would happen. It has also been reported that relapsing hyperparathyroidism by autografted parathyroid tissue may require extensive demolition of surrounding muscle tissue in addition to excision of hyperactive parathyroid tissue (8). Therefore, identification of hyperactive parathyroid tissue preoperatively is important.

Numerous examination medthods were used preoperatively or intraoperatively in localizing the hyperfunctional parathyroid lesions or predicting the presence of multiglandular disease (3, 9–11). There is widespread used of Technetium 99m methoxyisobutylisonitrile (^99m^Tc-MIBI) dual-phase planar imaging and single-photon emission computed tomography (SPECT) combined with computed tomography (CT)-SPECT/CT fusion imaging in PHPT and SHPT (12–14). It is used for the localization of hyeperfunction parathyroid glands in PHPT and significantly improved the outcomes of patients undergoing minimally invasive parathyroidectomy in PHPT and can also assist surgeons in selecting the lowest degree of PTG hyperplasia which is appropriate to autotransplantation in SHPT (15). However, there are few articles discussed about the differences between the ^99m^Tc-MIBI uptake in PHPT and SHPT. This study aims to evaluate the features of PHPT and SHPT, and the differences between them, by examining several biomarkers (PTH, alkaline phosphatase [ALP], phosphorus [P] and Ca), the volume of the tumors, and ^99m^Tc-MIBI uptake in dual-phase planar and SPECT/CT fusion images.

## Materials and methods

### Patients

**Figure 1:**
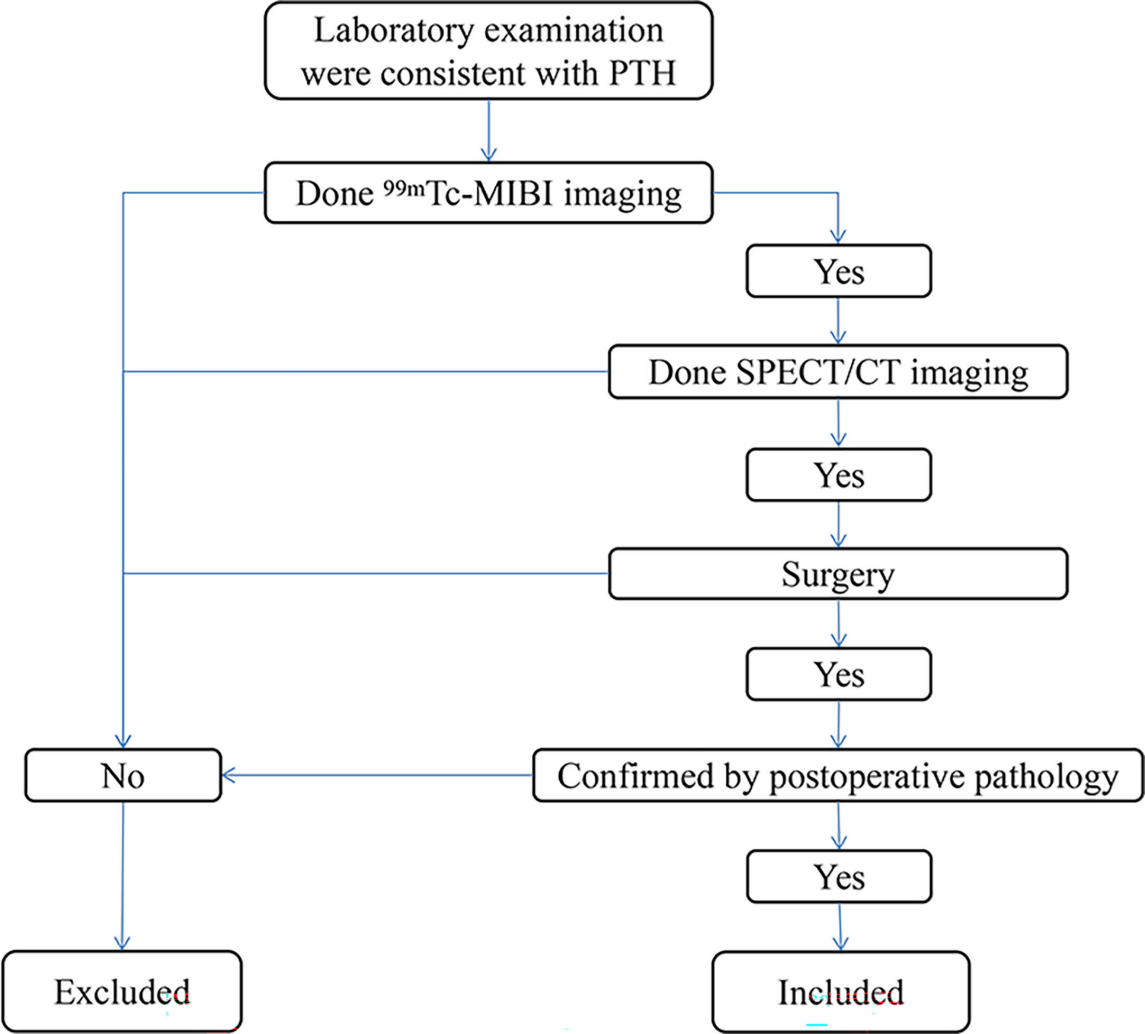
Inclusion and exclusion criteria roadmap.

Inclusion criteria: 1.The patient visited our hospital from 2015 to 2019 and had elevated PTH on laboratory examination. 2.The patient has underwent test ^99m^Tc-MIBI imaging. 3.The patient has underwent test SPECT/CT imaging. 4.The patient underwent parathyroid surgery and was confirmed by postoperative pathology. A total of 33 patients were included. (**Figure 1**). Chronic kidney disease (CKD) patients with hyperparathyroidism caused by dialysis and unable to be controlled by drug therapy were divided into SHPT group (14 patients in total) and the remaining patients were divided into the PHPT group (19 patients in total). Preoperative PTH, Ca, P and ALP were collected from the two groups. All resected parathyroid glands (PTGs) were measured, and the volume of each PTG was estimated using the following formula: *a × b × c* × π/6 cm^3^ (where *a*, *b*, and *c* are the dimensions of the gland in centimeters) (5, 6).

### Imaging examinations

Imaging was performed on a double-head gamma-camera equipped with a low-energy, high-resolution collimator (Discovery NM/CT 670, GE Healthcare). The camera was set at a 140 keV photo peak with a 20% energy window. Anterior neck images were obtained in 256 × 256 matrix size, gathering 500k counts per position. The patients received an intravenous injection of 740 MBq of ^99m^Tc-MIBI. Early-phase planar images were obtained 20 mins after the injection and delayed-phase planar images were obtained 2 h after the injection. Immediately following this, the SPECT/CT fusion images were obtained. The SPECT images included the neck and thorax. A 128 × 128 matrix was used, and images were obtained using 3° per step and 20 s per step over 360°. A CT scan was performed immediately after the SPECT imaging. The main CT parameters were 120 keV, 200 mAs, and a 2.5-mm slice thickness. The SPECT/CT fusion image data were analyzed on a workstation (GE healthcare), which provided transaxial, coronal, and sagittal slices using the SPECT, the CT, and the fused SPECT/CT data.

### Imaging analysis

The imaging results were evaluated using visual and semi- quantitative analyses by two experienced nuclear medicine physicians who were blinded both to the surgical results and the histopathology of the lesions. Positive ^99m^Tc-MIBI scans indicated a fixed concentration in neck or mediastinum on imaging with a parenchymal space-occupying lesion (independent soft tissue mass) in the corresponding position on CT imaging or a soft tissue mass in parathyroid area without ^99m^Tc-MIBI concentration in SPECT/CT. For semi-quantitative analysis that compared the positive results found on the dual-phase planar imaging and on the SPECT/CT imaging, a region of interest (ROI) was defined manually on the areas of increased ^99m^Tc-MIBI uptake indicating the presence of lesions in both types of imaging, and an identical ROI was identified on the contralateral side. The target-to-non-target ratio (T/NT) ratio was calculated using the following formula: average lesion count in the ROI/average contralateral tissue counts of ROI.

### Statistical analysis

Statistical analysis was performed using SSPS (version 24) software. Continuous data were expressed as mean ± standard deviation or median with interquartile range. The nonparametric Mann–Whitney test was used to compare the T/NT ratios, the levels of biochemical markers, and tumor volumes in the PHPT and SHPT groups. Spearman’s rank correlation coefficient was used to calculate the correlation between the T/NT ratio, the biochemical marker levels and the volume of the PTG. The T/NT ratios and tumor volumes were evaluated in relation to the tumors’ histopathology using the Kruskal–Wallis analysis of variation (ANOVA). In the case of multiple lesions, the tumor volume was calculated by summing the volumes of all the lesions. Statistical significance was set at *P* < 0.05.

## Results

### Patient characteristics and preoperative biochemical marker levels

Among the 19 patients with PHPT, there were nine men and 10 women, and the median age was 63 years. In this group, 18 patients had a raised serum PTH level which declined postoperative, and one patient had a normal serum PTH level; 10 patients had a raised serum ALP level, and nine patients had a normal serum ALP level; 17 patients had a raised serum Ca level, and two patients had a normal serum Ca level. All patients had a normal or low serum P level. Among the 14 patients with SHPT, there were 10 men and four women, and the median age was 34 years. The primary diagnosis of the SHPT patients was 5 chronic kidney disease and medicine had ever been used to control PTH level. In this group, all patients had a raised serum PTH level; 10 patients had a raised serum ALP level, and four patients had a normal serum ALP level; four patients had a raised serum Ca level, and 10 patients had a normal serum Ca level; 13 patients had a raised serum P level, and one patient had a normal serum P level. The median or mean serum levels of PTH, ALP, Ca, and P in PHPT and SHPT are shown in **Table 1**. According to the Mann–Whitney test, the median age in PHPT was higher than SHPT with significance [P=0.001]. The serum levels of both PTH and P were found to be significantly higher in the SHPT patients than in the PHPT patients (*P<*0.001, both). The serum level of Ca was found to be significantly higher in the PHPT patients than in the SHPT patients (*P*<*0.001*).

**Table 1:** Baseline characteristics of patients included in the study.

Variables	PHPT	N1	SHPT	N2	t/Z/χ^2^	P-value
Age (years)	63 (52-73)	19	34 (29-47)	14	-3.190	0.001
Sex (male/female)	10/9	19	4/10	14	1.910	0.167
Preoperative PTH (pg/mL)	334.50 (131.40-564.80)	19	1668.65 (751.67-2763.05)	14	4.025	<0.001
Preoperative ALP (IU/L)	125.00 (103.70-218.20)	19	279.70 (78.27-752.37)	14	0.984	0.341
Preoperative P (mmol/L)	0.82 (0.61-1.02)	19	1.94 (1.74-2.58)	14	4.773	<0.001
Preoperative Ca (mmol/L)	2.82 (2.69-3.35)	19	2.40 (2.15-2.60)	14	-4.408	<0.001
Early T/NT Ratio	1.51 (1.22-1.93)	19	1.46 (1.15-1.58)	23	-1.150	0.25
Delayed T/NT Ratio	1.34 (1.21-1.62)	19	1.30 (1.15-1.54)	23	-1.049	0.294
SPECT/CT T/NT Ratio	2.75 (2.20-3.84)	19	1.38 (1.13-2.61)	26	-3.171	0.002
Volume of PTG (cm^3^)	1.69 (0.65-2.93)	19	0.52 (0.16-1.42)	43	-3.306	0.001

PHPT, primary hyperparathyroidism; SHPT, secondary hyperparathyroidism; PTH, parathyroid hormone; P, phosphorus; Ca, calcium; ALP, alkaline phosphatase; BUN, blood urea nitrogen; Scr, creatinine; T/NT, target-to-non-target; PTG, parathyroid gland. N1: the number of PHPT group; N2: the number of SHPT group. Data are represented as median (25–75th percentile).

### Surgical and histopathological results

In total, 62 glands were resected in the 33 patients. In the 19 patients who had PHPT, 16 underwent traditional surgery and 3 underwent minimally invasive approch (MIAVP), 19 PTGs were resected and their histopathology was examined. This confirmed 14 cases of parathyroid adenoma and five cases of parathyroid carcinoma. Among the 14 patients with SHPT, two patients had one excised gland that indicated by preoperative imaging examination, one patient had two excised gland indicated by imaging examination, one patient had three lesions removed intraoperatively but pathological showed two parathyroid lesions, one patient had all three lesions found removed intraoperatively, two patients had four lesions resected intraoperatively but pathological showed three parathyroid lesions, two patients underwent subtotal parathyroidectomy, two patients had total parathyroidectomy with auto-transplantation and the rest three patients had total parathyroidectomy without auto-transplantation. 43 of the glands excised confirmed pathologically origined of the parathyroid gland. A subsequent histopathological examination confirmed the following diagnoses: parathyroid adenoma was found in 17 glands, parathyroid hyperplasia was found in 18 glands, and eight glands had features of both nodular hyperplasia and adenoma. According to the histopathological examination, the median volumes of the PTGs in the PHPT group and the SHPT group were 1.69 cm^3^ and 0.52 cm^3^, respectively (**Table 1**). A statistically significant difference was observed between the two groups (*P* = 0.001).

### Results from the parathyroid imaging examinations

In all 19 PHPT patients, the ^99m^Tc-MIBI dual-phase planar imaging yielded positive scintigraphic findings, and 19 lesions were detected in total, all of which were single focal lesions. All of these 19 lesions in planar imaging were also visible upon SPECT/CT fusion imaging. The dual-phase planar imaging detected 23 lesions in 13 out of the 14 patients with SHPT, with nine patients found to have a single focal lesion, and four patients found to have multiple lesions. However, the SPECT/CT fusion imaging in SHPT patients found 26 lesions with an increased uptake of ^99m^Tc-MIBI and eight lesions with no increased uptake of ^99m^Tc-MIBI. The results of the SPECT/CT fusion imaging suggested that of the 14 SHPT patients, three had a single lesion, five had two lesions, four had three lesions, and two had four lesions. One SHPT patient had a negative ^99m^Tc-MIBI dual-phase planar imaging result, but did have visible lesions upon SPECT/CT fusion imaging with no ^99m^Tc-MIBI uptake.

### Semi-quantitative analysis of technetium 99m methoxyisobutylisonitrile imaging

The T/NT ratios calculated based on ^99m^Tc-MIBI early-phase planar imaging in PHPT and SHPT were 1.51(1.22-1.93) and 1.46(1.15-1.58), respectively. The T/NT ratios calculated based on ^99m^Tc-MIBI delayed phase planar imaging in PHPT and SHPT were 1.34(1.21-1.62) and 1.30(1.15-1.54), respectively. The early-phase T/NT ratios were higher than the delayed-phase T/NT ratios for both the PHPT patients and the SHPT patients. When considering both phases, the T/NT ratios were higher in the PHPT patients than in the SHPT patients, but these differences were not statistically significant.

**Figure 2:**
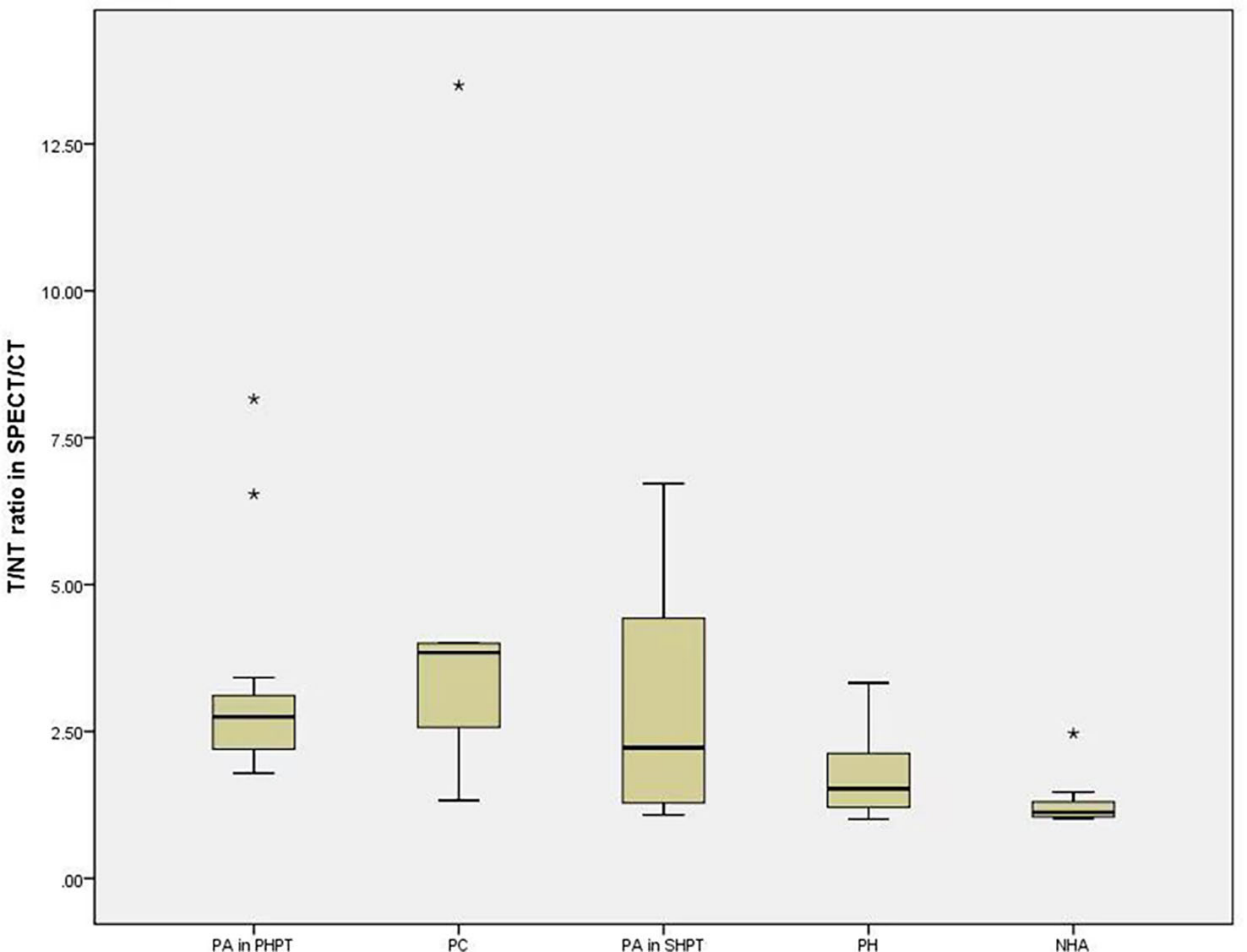
The difference of T/NT ratio in SPECT/CT fusion imaging among the subgroups. Boxplots indicate median value (black), first and third quartile range (box). Pairwise comparison by independent sample Kruskal-Wallis test, there was statistical significance between NHA and PA in PHPT (P=0.004). There was statistical difference between NHA and PTC (P=0.14). T/NT, target-to-non-target. PA in PHPT, parathyroid adenomas in primary hyperparathyroidism; PC, parathyroid carcinomas; PA in SHPT, parathyroid adenomas in secondary hyperparathyroidism; PH, parathyroid hyperplasia; NHA, nodular hyperplasia with adenoma. * indicates that the value is an outlier, which is far away from the third quartile line.

**Figure 3:**
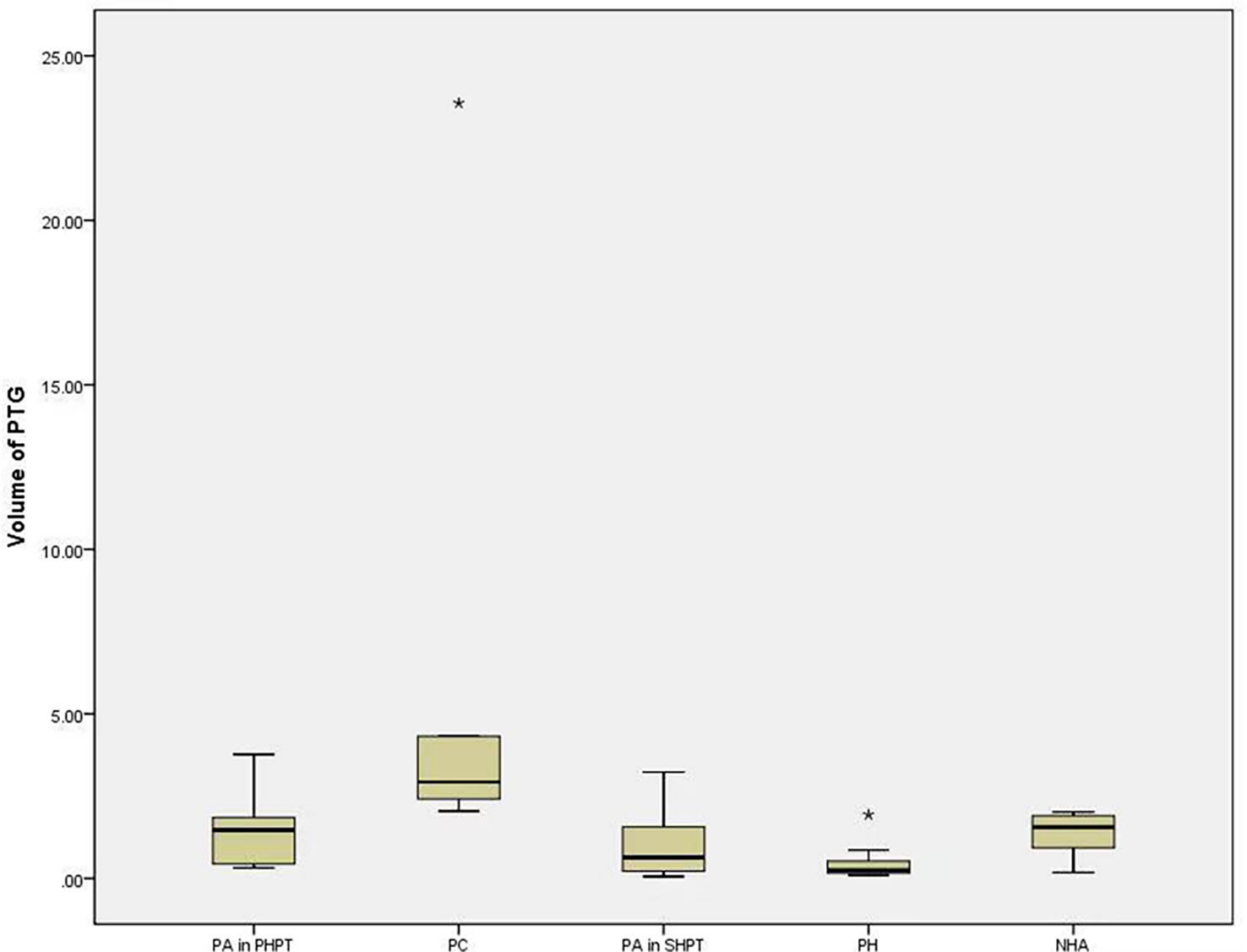
The difference of volume of PTG in subgroups. Boxplots indicate median value (black), first and third quartile range (box). Pairwise comparison by independent sample Kruskal-Wallis test, PH and PA in PHPT were statistically significant (P=0.023), PH and PC were statistically significant (P< 0.001), PA in SHPT and PC were statistically significant (P=0.025). PTG, parathyroid gland. PA in PHPT, parathyroid adenomas in primary hyperparathyroidism. PC, parathyroid carcinomas; PA in SHPT, parathyroid adenomas in secondary hyperparathyroidism; PH, parathyroid hyperplasia; NHA, nodular hyperplasia with adenoma. * indicates that the value is an outlier, which is far away from the third quartile line.

The median T/NT ratio of lesions found upon SPECT/CT fusion imaging in PHPT and SHPT was 2.75 and 1.38, respectively, and the difference between these was statistically significant (*P*=0.002). The 45 parathyroid lesions with positive SPECT/CT fusion imaging results were classified into five subgroups according to their histopathology: 14 parathyroid adenomas found in 14 PHPT patients (PA in PHPT), five parathyroid carcinomas found in four PHPT patients (PC), nine parathyroid adenomas found in five SHPT patients (PA in SHPT), nine instances of parathyroid hyperplasia found in five SHPT patients (PH), and eight instances of nodular hyperplasia with adenoma found in three SHPT patients (NHA). The Kruskal–Wallis ANOVA test indicated significant differences in the serum PTH, Ca, and P levels between the subgroups (*P* = 0.001, P<0.001, P<0.001,respectively. There was also a significant difference in the T/NT ratio of the SPECT/CT fusion images and the lesion volume between the subgroups (*P* =0.002, P<0.001, respectively). The lowest T/NT ratio was found in the NHA subgroup and the highest in PC subgroup. The largest lesion volume was found in the PC subgroup, and the smallest lesion volume was found in the PH subgroup (**Figures 2**, **3**).

## Discussion

The radiopharmaceutical ^99m^Tc-MIBI is liposoluble, intracellular, and cationic, and it accumulates in the mitochondria of viable cells by means of an electrochemical gradient due to the activity of respiratory chain (16). It is a radiopharmaceutical commonly used to perform parathyroid scintigraphy, and it can accumulate in abnormal PTG tissues, especially those that are rich in oxyphil cells (17). The sensitivity of ^99m^Tc-MIBI scan was 100% in oxyphil cell dominant PHPT patients and was 71.2% in chief cell and mixed cell-dominant PHPT patients (18). The study conducted by Cordes et al. (19) demonstrated that in 82% of ^99m^Tc-MIBI negative cases oxyphil cells were absent. The absence of oxyphil cells, with their large numbers of mitochondria, in PTGs probably lead to a decrease in the number of radiotracer binding sites resulting in negative ^99m^Tc-MIBI imaging (19). The PTG in true positive ^99m^Tc-MIBI imaging consisted predominantly of oxyphil cells and opposite in false negative ^99m^Tc-MIBI imaging (20). SHPT, a common complication of CRF, is characterized by parathyroid hyperplasia consisting mainly of chief cells (21). A meta-analysis showed the sensitivity of ^99m^Tc-MIBI plannar imaging in SHPT patients was 58% (16). In our study, the ^99m^Tc-MIBI dual-phase planar imaging in SHPT patients was positive in 13 out of 14 patients. The T/NT ratios of the ^99m^Tc-MIBI dual-phase planar images were lower in the SHPT patients than in the PHPT patients, but not to a statistically significant degree. The T/NT ratios in the SPECT/CT fusion images, eliminating overlapping effects, were significantly lower of the SHPT patients compared to PHPT. Adenomas were primarily present in the PHPT patients, while hyperplasia was primarily present in the SHPT patients. Parathyroid adenomas and parathyroid carcinomas are mainly composed of a mixture of chief and transitional oxyphil cells (22). Chief cells were found in all parathyroid hyperplasia lesions, and oxyphil cells were found in 67.9% of parathyroid hyperplasia lesions (23).

In our study, all the lesions in PHPT which were pathologically confirmed as adenomas and carcinomas were positive both on the dual-phase ^99m^Tc-MIBI planar imaging and the SPECT/CT fusion imaging. Among the 14 SHPT patients, 12 had multiple lesions, but not all of the lesions in SHPT were positive on the ^99m^Tc-MIBI dual-phase planar imaging. The SPECT/CT fusion imaging T/NT ratios were lowest in the NHA subgroups, and highest in the PC subgroup. These findings suggest that ^99m^Tc-MIBI may uptake less in SHPT than PHPT lesions. There are some studies about the oxyphil cells proportion of parathyroid lesions in the PHPT or SHPT (24, 25), but there is little studies directly compared the oxyphil cells proportion between PHPT and SHPT. The predominant cell type found in PHPT and SHPT lesions may account for the differences in the degree of visualization of different lesions on ^99m^Tc-MIBI dual-phase planar imaging.

Another factor that influences ^99m^Tc-MIBI accumulation in the parathyroid glands is the parathyroid lesion size (20). Histopathological examination of the resected glands after surgery revealed that the mean volume of the parathyroid lesions was significantly larger in the PHPT group than in the SHPT group in our study. Other studies have also reported the weight and volume of parathyroid lesions. In PHPT, the median weight of an adenoma was found to be 3.0 g-4.1g (26, 27). While the mean mass of lesions in SHPT was found to be 0.91g (28) and the mean volume of a PTG was 838 ± 939 mm^3^ in a study with CKD), which is consistent with the present study (29). Elsewhere, in a study of 17 patients, the median weight of parathyroid adenomas gland was larger than of hyperplastic glands with difference (30). The smallest volume in our study was evident in the PH subgroup. Oxyphil cells are larger than chief cells histologically (12–20 and 6–8 μm, respectively) (31). It had a trend towards larger size and weight when the percentage of oxyphil cells >75% in PHPT lesions (25). The volume of lesions decreased as the number of lesions increased (32). Multiple parathyroid lesions are often involved in patients with SHPT. The growth of PTG was stopped by the active form of vitamin D (calcitriol) in chronic renal failure (CRF) patients with comorbid SHPT (33). Most SHPT patients in CKD and SHPT receive vitamin D treatment. Considering the above factors, the difference of volume between PHPT and SHPT is related to oxyphil cells components and disease characteristics.

^99m^Tc-MIBI SPECT/CT sensitivity is significantly lower in multiple gland disease (MGD) than single-gland disease (SGD) may due to the reason that MGD usually is due to hyperplasia and SGD usually is due to adenoma (32). All the PHPT patients had a single parathyroid lesion identified upon ^99m^Tc-MIBI dual-phase planar imaging were confirmed by surgery and pathology. Among the 13 SHPT patients with positive ^99m^Tc-MIBI dual-phase planar imaging result, 11 patients had multiple lesions. In addition, the SPECT/CT fusion imaging found a further eight lesions in these 13 SHPT patients that had no increased ^99m^Tc-MIBI uptake. Parjeet Kaur et al. (26) suggested that the misdiagnosis of MGD as a single adenoma(SA) on ^99m^Tc-MIBI scans may be the result of an increased focus on a single enlarged gland, which leads to other small lesions being missed in the context of MGD. The higher rate of negative ^99m^Tc-MIBI dual-phase planar imaging results in SHPT compared with PHPT may be related to multi-gland hyperplasia.

The study by Min Zhang et al. (34) showed the retention rate of ^99m^Tc-MIBI in PC lesions was significantly higher than that in benign lesions, and the diameter of PC was larger than adenoma (3). In consist with them, the highest T/NT ratio and the largest volume is both in PC subgroup in our study. Among the pathologic lesions responsible for primary hyperparathyroidism, include adenoma, atypicaladenoma, double adenoma, multigland hyperplasia, and rarely carcinoma, 0.5-2% is parathyroid carcinoma (21). In this study, all 5 cases of parathyroid adenocarcinoma received treatment due to PHPT-related symptoms and were pathologically confirmed as cancer after surgery. We selected ^99m^Tc-MIBI positive patients with post-operative pathology from suspected HPT patients, so the total number of patients selected was small, leading to selection bias.

There was a significant difference between the mean age of PHPT and SHPT patients in our study. The median age of PHPT patients was 63 year and the median age of SHPT patients was 34 years with significant difference in our study. This result is consistent with the reported mean age of PHPT patients and the group age of SHPT mentioned in the study (2, 35, 36). The age of SHPT patients become younger with the degree of disease (37). With the growth of age or pathological state, the number of eosinophils from the degeneration of the master cells in parathyroid tissues gradually increases (38, 39). In a study of SHPT, the number of focal eosinophilic cells increased with age and the mean age in higher oxyphil cell proportion group was older than in lower group (24). We speculated the difference age between PHPT and SHPT patients may be related to the oxyphil cells.

Our study is limited by its relatively small number of patients and retrospective design which had not analyzed the chief cell and oxphil cell components in the resected lesions. Therefore, future prospective large cohort study needed for analyzing the difference that the uptake of ^99m^Tc-MIBI and the oxphil content in parathyroid lesions between PHPT and SHPT patients.

In conclusion, the proportion of patients who had a positive result on the ^99m^Tc-MIBI dual-phase planar imaging was higher in the PHPT group than in the SHPT group, the T/NT ratios on the ^99m^Tc-MIBI dual-phase planar and SPECT/CT fusion imaging were higher in the former group and the volume of parathyroid lesions involved in SHPT was also smaller than in PHPT. Therefore, it is recommended to combine other technology when ^99m^Tc-MIBI imaging is negative in patients with SHPT and it should be aware the possibility of cancer when abnormal concentration and large lesions observed in PHPT patients.

## Data availability statement

The original contributions presented in the study are included in the article/supplementary material. Further inquiries can be directed to the corresponding author.

## Ethics statement

The studies involving human participants were reviewed and approved by Ethics committee of Shanxi Bethune Hospital, Shanxi Academy of Medical Sciences, Tongji Shanxi Hospital, Third Hospital of Shanxi Medical University.

## Author contributions

Conception and design of the research: YW and WZ. Acquisition of data: YL and NL. Analysis and interpretation of the data: YW and NL. Statistical analysis: YL. Writing of the manuscript: YW. Critical revision of the manuscript for intellectual content: WZ. All authors contributed to the article and approved the submitted version.
